# Lived Experience of Patients Living with Glaucoma: A Qualitative Study

**DOI:** 10.31729/jnma.9041

**Published:** 2025-06-30

**Authors:** Anjani Kumar Singh, Krishna Kumar Thakur, Sumit Kumar Mishra, Binita Pandit, Rajiv Ranjan Karn

**Affiliations:** 1 Department of Glaucoma and Out Patient Department, Biratnagar Eye Hospital, Biratnagar, Morang, Nepal

**Keywords:** *glaucoma*, *lived experience*, *qualitative research*

## Abstract

**Introduction:**

Glaucoma is the leading cause of irreversible blindness in the world. There are limited qualitative study focusing on patients' experience of glaucoma. The aim of the study was to explore the lived experiences of patients living with glaucoma.

**Methods:**

A qualitative study was conducted involving eight patients from January 2024 to June 2024 at glaucoma department of an eye hospital after approval from the institutional review committee (Reference number: BEH-IRC/093/024), using in-depth interview. Semi-structured interviews were carried out with selected participants until data saturation was achieved. The interviews were audiorecorded, transcribed verbatim, and analyzed using thematic analysis. Patients receiving glaucoma treatment for more than five years and willing to participate were included in the study.

**Results:**

Out of the eight participants, seven were male with mean age of 43.12±12.86 years. Based on the interviews, seven main themes were identified. These include experiences and ideas about the moment they first noticed the condition, their emotional response after being diagnosed, a sense of relief after receiving accurate information, how they manage the disease, their sources of information, changes in their work life, and the involvement of family members in attending regular glaucoma checkups.

**Conclusions:**

This study explored the experiences of patients living with glaucoma. Findings showed that the patients with early diagnosis, proper education with counseling, proper treatment and regular follow up preserved their visual function.

## INTRODUCTION

According to World Health Organization (WHO), glaucoma is the second leading cause of irreversible blindness worldwide.^1-4^ Glaucoma is often called the "silent thief of sight.^5^ With timely diagnosis, 98% of vision loss can be prevented.^6^ A population-based survey from Nepal found overall prevalence of glaucoma to be 1.9%.^7^

Glaucoma patients need lifelong monitoring and proper medications, as poor treatment adherence can lead to blindness. Studies focusing on the experiences of patients living with glaucoma are crucial.^8-10^ It is well recognized that glaucoma affects daily life through visual deterioration which subsequently brings reduced quality of life and results in high healthcare costs.^11^ However, the importance of involving patient's experiences during the management of vision impairment is not well explored.

There is a paucity of literature on the qualitative experiences of glaucoma patients in Nepal. This study aimed to explore the life experiences of glaucoma patients visiting an eye hospital.

## METHODS

This was a phenomenological study conducted among the patients having glaucoma and ongoing treatment at Biratnagar Eye Hospital, Morang, Nepal. The study was conducted between January 2024 and June 2024, using consecutive sampling methods after approval from the institutional review committee (IRC) of Biratnagar Eye Hospital (Reference number: BEH-IRC/093/024). The study participants over 20 years of age, receiving treatment for more than five years were recruited from Biratnagar Eye. Informed written consent was obtained from all participants.

In-depth interviews using predetermined open-ended questionnaire were conducted with patients living with glaucoma to explore their socio-demographic backgrounds, understanding of the disease, and personal experiences throughout their journey with the condition. Qualified ophthalmic assistant working in the glaucoma department experienced in qualitative research conducted the interviews in a quiet, undisturbed setting, in language comfortable to patients. The study sample was selected through consecutive sampling, with eight participants meeting saturation. The inclusion criteria were those glaucoma patients who had been under treatment for glaucoma for more than five years. Individuals were excluded if they were unwilling to participate or had vision deterioration unrelated to glaucoma. The interviews, lasting between 12 to 35 minutes, were recorded using a tape recorder and kept safely. Participant confidentiality was maintained throughout the process. COREQ guidelines were followed.^12^

Following the completion of the interviews, the data was transcribed to capture the participants' responses accurately. Once the data was transcribed, it underwent a thorough analysis using a "read and re-read" process. This method involved repeated readings of the transcripts to identify key patterns, themes, and insights related to the participants' experiences with glaucoma. Thematic analysis was employed to analyze the data, which involved identifying recurring themes or concepts that emerged from the participants' responses. This process was done manually with the assistance of the research team.

Once the initial themes were developed, a member of the research team, typically an expert in qualitative research, was invited to review the themes and provide feedback. The final themes were prepared for reporting and further interpretation within the study context. Glaucoma diagnosis and severity were categorized by following system proposed by Mills et al.^13^

## RESULTS

A total of eight participants completed the interviews, with an average age of 43.12±12.86 years. Out of the total participants, 7 (87.50%) were male, all were married, 5 (62.50%) had attained higher-level education, primary open-angle glaucoma was 7 (87.50%), and 5 (62.50%) had severe glaucoma. The mean duration since diagnosis with glaucoma was 14.62±11.61 years (Table 1).

**Table 1:** Demographic characteristics of participants with glaucoma (n=8).

Characteristics	n(%)
**Gender**	
Male	7(87)
Female	1(12)
**Marital Status**	
Married	8(100)
**Education**	
Illiterate	1(12)
Secondary Education	2(25)
Bachelor's and above	5(62)
**Glaucoma Type**	
Primary Open Angle Glaucoma (POAG)	7(87)
Congenital Glaucoma	1(12)
**Degree of Glaucoma**	
Moderate	3(37)
Severe	5(62)
Duration since Diagnosis (mean±SD)	15±12 (years)
Below 15 years	6(75)
Above 15 years	2(25)

**Theme 1: First noticed this eye disease.**

Participants were diagnosed with glaucoma when they visited the hospital after having different eye problems. These symptoms included a blurred vision, excessive watering of the eyes, and persistent itching. These signs initially seemed minor, but they sought medical attention as they worsened. Upon further examination, it was revealed that these seemingly common eye problems were early indicators of glaucoma, a condition that can cause irreversible vision loss if left untreated.

One of the male participants of age 40 years decided to have his eyes checked while he was at the hospital accompanying his mother for her eye treatment. Despite not experiencing any noticeable symptoms or issues with his vision, the screening revealed that he had glaucoma. Participants describe their experiences as follows:

*"At the age of 13, my vision deteriorated, and I couldn't see clearly, unaware I had glaucoma. My teacher noticed something unusual about my eyes. As a careless child, I didn't realize I had an eye problem. Due to my family's financial struggles, my teacher collected money for my treatment. After seeking treatment, I was diagnosed with glaucoma during my first visit to Eye Hospital," (Participant 1)*

*"I randomly had my eyes checked at two or three places despite having no symptoms, but they didn't detect glaucoma. I first learned I had it at the Eye Hospital when I accompanied my mother for her treatment and decided to get examined myself. After the check-up, I discovered I had glaucoma, while my mother did not," (Participant 5)*

This diagnosis highlighted the silent progression of the disease, which can develop without warning signs and often goes unnoticed until significant damage has occurred. All participants reported being diagnosed with glaucoma after undergoing an eye examination at the hospital, which revealed elevated Intra Ocular Pressure.

**Theme 2: Feeling, when diagnosed with Glaucoma**

Most of the participants reported the emotional and practical challenges faced by individuals when diagnosed with glaucoma as they were worried about the possibility of becoming blind. Living with impaired vision can lead to feelings of isolation, anxiety about the future, and limitations in career and personal opportunities. It emphasizes the profound impact of the condition on daily life, aspirations, and mental well-being.

*"When I found that I had glaucoma, with nerve damage and deteriorating vision, I felt devastated and believed that I might lose sight in that eye permanently," (Participant 5)*

*"I felt isolated because my impaired vision set me apart from those with good eyesight. I considered moving abroad for opportunities, but my condition hindered my plans, including pursuing a computer job or driving. I worry about the possibility of becoming blind and what the future might hold, which naturally causes anxiety and concern," (Participant 1)*

**Theme 3: Feeling After Receiving Correct Treatment**

All participants were satisfied after receiving appropriate treatment for glaucoma. They highlighted that early detection and timely treatment significantly eased their management of the disease. Addressing glaucoma in its initial stages played a crucial role in preventing blindness, demonstrating the effectiveness of prompt and proper care in preserving vision and overall eye health.

*"After treatment, my right eye's vision improved by 95%. During a follow-up, glaucoma was detected in my left eye too, requiring surgery by the same doctor. The surgery resulted in a 40% improvement. I am satisfied with the care I received, having visited the hospital thrice a year. I believe my vision is good, and my eyes are healthy, likely free from glaucoma. I think I am free from glaucoma," (Participant 2)*

*"If I hadn't received timely treatment, I might have gone blind. Treating the condition in its early stage prevented blindness. Thanks to the treatment, I can see things clearly, and my eyes are now in good condition," (Participant 3)*

**Theme 4: Managing the Condition by Using Eye Drops and Attending Regular Follow-Up Exams**

All participants reported consistently using their eye drops and attending follow-up appointments as directed by their ophthalmologist. However, three participants mentioned that the high cost of the medications imposed an economic burden. A few others admitted to occasionally missing to the usage of the drops. Despite these challenges, all participants recognized the importance of regular eye drops use and timely follow-ups in maintaining their vision.

*"I believe that if I don't use the prescribed drops, my vision may deteriorate, so I've never missed a dose. I use them consistently and always attend my follow-up appointments on time. I think regular check-ups and consistent medications are essential for protecting my vision and maintaining overall eye health," (Participant 7)*

*"When I was 20, if I hadn't taken these issues seriously, recognized the risks, or sought timely treatment, I would have lost my vision within 2 to 4 years due to glaucoma. I realized that getting the right treatment and regular follow-ups was crucial to preserving my vision. Maintaining my vision for 40 years has been a significant achievement in managing glaucoma." (Participant 3)*

**Theme 5: Source of information about Glaucoma**

Most participants learned about glaucoma primarily from their ophthalmologist or online sources after their diagnosis. A few did not seek additional information and relied solely on their prescribed medication. Generally, participants understood that glaucoma affects the optic nerve by increasing ocular pressure, and if left untreated, it could lead to blindness. They stated that awareness was crucial for their understanding of the importance of their treatment and helped them manage the condition more effectively.

*"I had never heard of glaucoma before, so I was worried when I was diagnosed with glaucoma. After school, I received a mobile phone, I started researching interviews with doctors from Nepal on glaucoma topics. Then I started to research and check on the internet. I came to know that vision loss from glaucoma cannot be restored, the remaining vision can be protected. I studied the various types of glaucoma, such as congenital, open-angle, and closed-angle glaucoma. I've also learned about the surgical options and how these procedures are carried out. For those who cannot rely on medications, surgery is necessary, and there are also laser treatments available. With this knowledge, managing glaucoma has become much easier for me," (Participant 1)*

**Theme 6: Occupational changes due to glaucoma.**

Most of the participants faced problems in their profession and in carrier growth due to poor vision and deterioration in the visual field. Some of them lost their job and some of them had to change their profession.

*"As I am a railway driver now, I am staying at home as I lost my current job due to deterioration in my visual field. They said that my visual field had deteriorated, placing me in the Driving Safety category. In this category, I cannot get a job." (Participant 5)*

**Theme 7: Urging Family Members to Attend Regular Glaucoma Checkups**

Four Participants mentioned that they had their family and siblings' eyes examined because glaucoma is a hereditary disease. The other half said they haven't had their family and siblings' eyes checked yet because they haven't developed any symptoms. A few participants also shared information about glaucoma with their relatives and friends to encourage timely eye examinations.

*"I have also had my son's eyes checked because I am aware that it is a genetic condition that could pass on to him. I took him to hospital for an eye examination, and fortunately, everything was normal," (Participant 1)*

*"My family and siblings never had eye checkups since they didn't experience any eye problems. I was unaware that this condition was genetic and could be passed on to my children and siblings. I will ensure that my children and siblings undergo eye checkups, as glaucoma affects our eyes gradually and unexpectedly," (Participant 6)*

## DISCUSSION

This study explored life experiences of patients living with glaucoma. Many participants were diagnosed with the condition during hospital visits prompted by blurred vision, watering, and itching. The findings highlighted that several participants visited due to various symptoms related to their eyes, few were diagnosed with glaucoma despite having no symptoms at all. Similarly, a study conducted by Iyigun E et.al revealed that most participants initially underwent eye examinations for reasons unrelated to glaucoma.^8^ They sought medical attention due to severe headaches, ocular pain, and changes or reductions in vision. Elevated ocular pressure was identified during these visits, leading to their eventual diagnosis.^14^ Similarly, A study conducted in the UK by Prior M.et al. revealed that a late diagnosis of glaucoma is related to not receiving routine eye examinations.^9^

The current study showed that the majority of participants expressed emotional and practical difficulties upon being diagnosed with glaucoma, particularly concerning the fear of potential blindness. Coping with impaired vision often results in feelings of isolation, anxiety about the future, and restrictions on both career and personal opportunities. The study highlights the significant effects of the condition on daily life, future goals, and mental well-being. Likewise, other studies have found that glaucoma disrupts daily life and can significantly affect activities, social status, and personal ambitions.^11, 14, 15^

In this study, participants sought information about the condition from their doctor and the internet after receiving a glaucoma diagnosis. Similarly, a study conducted in Gujarat found that the most common sources of glaucoma information were radio programs, reading materials, and the Internet.^11^ However, a study conducted in the UK by Abdull M. M. et.al, indicated that patients learned about glaucoma from hospital staff after diagnosis.^15^ Most participants in this study experienced shifts in their careers as a result of glaucoma.

The majority of participants in this study emphasized the importance of regular eye drops for managing glaucoma. Despite facing financial challenges, they consistently used the drops and attended follow-up eye exams on time. Likewise, a study conducted by Iyigun et.al. revealed that all participants recognized the significance of using eye drops and the necessity of attending follow-up eye examinations.^8^ In contrast, a study by Shah J, revealed an issue with administering eye drops, as patients had not received proper guidance on how to apply the drops in the hospital.^11^

In this study, participants expressed concern about their siblings and relatives potentially having glaucoma, which aligns with findings from other studies, as glaucoma has a genetic component.^9^ Due to this, participants encouraged their family members to undergo eye examinations. However, several participants mentioned that they had not yet arranged for their family members or siblings to have their eyes checked, as minimal symptoms had appeared. Some common findings reported by other researchers were not observed in our study, possibly because most of our patients had a long history of the disease and may have experienced recall bias. The study's findings may be limited by both time constraints during interviews, which restricted opportunities for in-depth responses, and some participants' reluctance to openly share emotional experiences, potentially affecting the comprehensiveness of the collected data

## CONCLUSION

This study explored the experiences of patients living with glaucoma after diagnosis and during treatment. As all of the participants had glaucoma, they were attending regularly for glaucoma follow- up examinations. All the participants responded well to treatment, likely because they had access to information about glaucoma, received positive counseling, and had trust in their treatment, which helped maintain their visual function effectively. It is unequivocal that continuous treatment and follow-up helps to prevent vision loss associated with glaucoma.
